# Estimating the workload associated with symptoms-based ovarian cancer screening in primary care: an audit of electronic medical records

**DOI:** 10.1186/s12875-014-0200-y

**Published:** 2014-12-12

**Authors:** Anita WeyWey Lim, David Mesher, Peter Sasieni

**Affiliations:** Centre for Cancer Prevention, Wolfson Institute of Preventive Medicine, Queen Mary University of London, Charterhouse Square, London, EC1M 6BQ UK

**Keywords:** Ovarian cancer, Symptoms, Screening, General practice, Early diagnosis

## Abstract

**Background:**

Ovarian cancer is the most lethal gynaecological malignancy in the United Kingdom (UK). Studies have found that many women with ovarian cancer have symptoms for several months before diagnosis. Using a symptoms-based tool to diagnose ovarian cancer (OC) earlier is appealing, but may increase general practitioner (GP) workload because the symptoms are typically vague and non-specific. This study aimed to provide estimates of the GP workload associated with offering symptoms-based ovarian cancer screening.

**Methods:**

A cross-sectional analysis of electronic records from four general practices in England, UK. We downloaded anonymous data on women aged 45–74 who consulted over one week to estimate the proportion who would be offered ‘screening’ according to the UK National Institute for Health and Care Excellence (NICE) guidelines and a symptoms index (Index 2) over one year. We used previous consultations (censoring women with no prior symptom at the date of their last recorded consultation) to estimate the proportion of women presenting with a new (not recorded in previous 12 months) NICE symptom each year.

**Results:**

Data were obtained from 19,558 women. The proportion presenting over one week varied between practices (5%-14%), however, the proportion with an OC symptom was similar (17% overall). Over one year, an estimated 51.8% (95% CI 44.0%-59.7%) would present with an OC symptom, 26.6% (95% CI 19.3%-35.1%) with a NICE symptom and 20.3% (95% CI 13.7%-28.5%) with an Index 2 symptom. Each year, an estimated 11.9% (95% CI 5.0%-18.3%) of women would present with a new NICE symptom.

**Conclusion:**

One in two women aged 45–74 present to primary care at least once a year with an OC symptom, 11.9% with a new NICE symptom. This would be comparable to 2 to 8 yearly screening (depending on what symptoms triggered testing).

## Background

Ovarian cancer is the most lethal gynaecological malignancy in the UK causing around 4,200 deaths per year [1]. The majority of women are diagnosed with advanced stage disease and the lack of an effective screening mechanism means that diagnosis relies on symptomatic presentation. Numerous retrospective studies [2-13] have shown that women have symptoms for many months before ovarian cancer diagnosis. This has led to interest in using symptoms to prompt investigations which could hopefully facilitate earlier stage diagnosis. This would be a form of targeted ‘screening’ in which women presenting to primary care with symptoms suggestive of ovarian cancer would be offered a blood test (e.g. CA125 or any future biomarker) and/or pelvic ultrasound. The challenge is that ovarian cancer is rare, and the symptoms are relatively common with non-malignant conditions in postmenopausal women (in whom >80% of ovarian cancers occur).

In the UK, NICE (National Institute for Health and Clinical Excellence) guidelines for ovarian cancer recommend CA125 testing for persistent symptoms of abdominal distension, feeling full quickly/loss of appetite, pelvic/abdominal pain and urinary frequency or urgency [14]. Urgent investigation for ovarian cancer is only recommended for women with suspected ascites or a palpable mass. In 2012, primary care physicians (GPs) in England were given increased access to non-obstetric ultrasound with the express purpose of aiding ovarian cancer diagnosis [15]. However, there are few data on the prevalence of ovarian cancer-like symptoms in women presenting to primary care or in the general population. Also, because of the growing demands on primary care, activities that could increase GP workload need careful assessment.

Electronic patient records (EPR) enable large amounts of data to be obtained with a simple download. The aim of this study was to use electronic medical records to provide an unbiased estimate of the GP workload associated with offering symptoms-based ovarian cancer screening.

## Methods

### GP practices

We identified potential GP surgeries in London, Newbury, Wokingham and Bracknell via research contacts and collaborators. Of those willing to participate we only included those with a list size of at least 5500, that had been using EMIS (Egton Medical Information Systems one of the main software suppliers to UK general practices) LV (Limited Version) for at least one year, had at least one female GP (in case women are less likely to report gynaecological symptoms to a male GP), and no unusual demographic composition. The threshold for list size was based on the average of 5891 patients for England set by the 2004 General Medical Services contract [16].

### Study population

Only anonymised data were used in the study, therefore ethics approval was not necessary. We used EMIS to download data on permanently registered women aged 45–74 years who presented over one week. Women whose records showed that they had a history of ovarian cancer or were actively receiving treatment for a malignancy (except for hormonal treatment only) were excluded.

### Data collection

Downloads took place between November 2007 and May 2008. We developed a search strategy in EMIS LV which downloaded data on women aged 45–74 who consulted over one week. Data included the woman’s age, medical history and details of the most recent previous consultation entries up to a maximum of 10. The week examined preceded the date of download by one day to allow time for after-hours and home visits to be entered.

We also collected the following details from each practice:

- List size (including breakdown of age and sex)
- Total number of women aged 45–74 in the practice
- Number of GPs
- Full-time or part-time work status of each GP
- Total number of women registered with the practice

### Data cleaning

Consultation data in EMIS include entries that are not actual consultations such as incoming letters, administrative notes and laboratory test results. For each consultation entry we determined who was seen/encountered (from the name of the person who made the entry; eg a doctor or receptionist’s name), the consultation location (from the consultation type field; eg “GP surgery”, “path lab”). Consultation entries were then limited to those that were with a GP or a nurse and in-person (GP surgery or home visits) or over the telephone.

The same researcher (AWL) extracted symptoms from the consultation free text and clinical codes. Extracted symptoms were then coded as relevant or not relevant to ovarian cancer according to a pre-defined list of symptoms most commonly reported by women with ovarian cancer [2-10]. Symptoms included pelvic or abdominal pain or discomfort; abdominal bloating; increased abdominal size/abdominal distension; abdominal lump; indigestion; constipation; diarrhoea; nausea or vomiting; other gastrointestinal symptoms (eg wind, change in bowel habit); loss of appetite; weight loss, fatigue; urinary frequency or urgency; postmenopausal bleeding; and vaginal discharge. Vaginal discharge is not usually considered to be a typical ovarian cancer symptom, but was included because we found it to be independently associated with ovarian cancer in a case–control study [12]. A systematic coding frame (compiled with the input of two gynaecologists: Professor Usha Menon and Dr Aarti Sharma) was used to ensure symptoms were classified consistently.

Details of co-morbidities (type and date diagnosed/recorded) were obtained from the women’s medical history summary data.

### Analysis

We estimated the proportion of women who would be offered targeted ‘screening’ in GP primary care over one week under two different scenarios. The first used NICE guidelines which recommend CA125 testing in women who have any one of persistent abdominal distension, feeling full (early satiety) and/or loss of appetite, pelvic or abdominal pain or increased urinary urgency and/or frequency, on a persistent or frequent basis particularly more than 12 times per month [14]. We applied these criteria to our data except for symptom frequency as this information was not available in the GP notes. The second used a symptoms index (Index 2) that we developed in a previous study [12]. Women are considered positive on Index 2 if they have any one of pelvic abdominal pain or discomfort; loss of appetite; increase in abdominal size; able to feel a lump in the abdomen; or vaginal discharge. We also considered women who had two or more Index 2 symptoms given that the positive predictive value of individual symptoms is low [17].

Targeted ovarian cancer ‘screening’ is unlikely to be triggered by longstanding symptoms (unless there is worsening or change). Therefore, we also estimated the proportion of women who presented with a NICE guideline symptom that was new in the previous 12 months. The time period covered by downloading the last 10 consultation entries varied between women (median 6.0 months inter-quartile range 2.8-11.5). To use all available data for each woman we used the Kaplan-Meier method to estimate the proportion of women with a NICE guideline symptom in the reference week who had presented previously with that symptom as a function of time. From this we estimated the proportion who had at least one NICE guideline symptom for which they had not presented in the previous year (ie ‘new’). We multiplied this by the proportion of registered women aged 45–74 who presented with a NICE symptom to calculate the proportion (p) who would present during the week with a NICE guideline symptom that was new in the last year. The Delta method was used to calculate the variance to obtain confidence intervals for our estimate of NICE guideline symptoms new in the last year (taking into account the Kaplan-Meier estimate).

We calculated the proportion of women aged 45–74 who presented during the reference week with symptoms with 95% confidence intervals (for proportions) for women with:

- Any ovarian cancer symptom
- Any NICE guideline symptom
- Any Index 2 symptom
- Any ‘new’ NICE guideline symptom
- At least two Index 2 symptoms

We approximate the proportion of all women aged 45–74 that would be offered testing over one year according to different symptom categories using the equation:

$$ 1-{\left(1-p\right)}^{52} $$

where *p* is the proportion of women to be offered testing in a single week. Confidence intervals (95%) were calculated using the same equation and were based on the binomial distribution.

## Results

A total of seven practices were approached of which five agreed to participate. Of these, one was excluded because the list size was too small (below 5500).

The combined list size was 38,921, of whom 5737 were women aged 45–74. Table 1 shows GP practice details and the number of women aged 45–74 who consulted during the reference week for each practice and overall. Although the proportion of registered females in the target age range at each practice was broadly comparable between practices (11%-17%), the proportion that consulted during the week varied considerably (e.g. almost 3 times as many women presented at Chrisp Street (14%) than at Boundary House and Woosehill practice (both 5%)). Of the women who consulted during the week the proportion who reported any symptom was similar across practices. However, again there was almost a 3-fold difference between practices in the proportion of all women aged 45–74 who presented with an ovarian cancer symptom during the week (0.8% (Woosehill) cf. 2.2% (Chrisp Street).

**Table 1:** **Details of GP practices and women aged 45–74 who presented during the reference week**

	**Chrisp St**	**Woosehill**	**Northcroft**	**Boundary**	**TOTAL**
List size	11504	10652	8928	7837	38921
Total number females	5645 (49%)	5388 (51%)	4503 (50%)	4022 (51%)	19558 (50%)
Women aged 45-74	1210 (11%)	1809 (17%)	1457 (16%)	1261 (16%)	5737 (15%)
GPs at practice					
Full-time	1	3	3	3	10
Part-time	11	2	2	1	16
Total	12	5	5	4	26
Women aged 45–74 who presented during the week					
with GP	133	56	124	38	351
with Nurse	50	36	45	24	155
Total (GP or nurse)	168* (14%)	85 (5%)	163^†^ (11%)	59 (5%)	475^‡^ (8%)
Proportion of presenting women who had ≥1 symptom during the week	112/168 (66.7%)	50/85 (58.8%)	93/163 (57.1%)	38/59 (64.4%)	293/475 (61.7%)
Proportion of all women aged 45–74 who presented with ≥1 ovarian cancer symptom during the week	27/1210 (2.2%)	14/1809 (0.8%)	28/1457 (1.9%)	11/1261 (0.9%)	80/5737 (1.4%)

^*^Excludes one woman with ovarian cancer.

^†^Excludes one woman with ovarian cancer and two women receiving treatment for other cancers.

^‡^Excludes two women with ovarian cancer and two women receiving treatment for other cancers.

Table 2 shows the estimated proportion of women who presented at each practice that would be offered testing in one week based on different criteria. According to the basic testing scenario of any ovarian cancer symptom, 16.8% of women aged 45–74 who present over one week would be ‘screened’ (equivalent to 1.4% of all women in the age group each week). It is much less common to have two or more Index 2 symptoms: 0.8% of women who present over one week had at least two Index 2 symptoms.

**Table 2:** **Estimated proportion of women presenting during the week who would be offered symptoms-based ‘screening’ according to different testing thresholds**

	**Chrisp Street**		**Woosehill**		**Northcroft**		**Boundary**		**TOTAL**	
	**(n =168)**		**(n =85)**		**(n =163)**		**(n =59)**		**(n =475)**	
	**n (%)**	**95% CI**	**n (%)**	**95% CI**	**n (%)**	**95% CI**	**n (%)**	**95% CI**	**n (%)**	**95% CI**
Any OC symptom	27 (16.1%)	10.9, 22.5	14 (16.5%)	9.3, 26.1	28 (17.2%)	11.7, 23.9	11 (18.6%)	9.7, 30.9	80 (16.8%)	13.6, 20.5
Any NICE symptom*	13 (7.7%)	4.2, 12.9	4 (4.7%)	1.3, 11.6	11 (6.7%)	3.4, 11.8	3 (5.1%)	1.1, 14.1	31 (6.5%)	4.5, 9.1
Any Index 2 symptom^†^	11 (6.6%)	3.3, 11.4	3 (3.5%)	0.7, 10.0	8 (4.9%)	2.1, 9.4	3 (5.1%)	1.1, 14.1	25 (5.3%)	3.4, 7.7
≥2 Index 2 symptoms	2 (1.2%)	0.1, 4.2	0 (0%)	0.0, 4.2	2 (1.2%)	0.1, 4.4	0 (0%)	0.0, 6.1	4 (0.8%)	0.2, 2.1

OC = ovarian cancer, NICE = National Institute for Health and Clinical Excellence.

*Any one of persistent abdominal distension, feeling full (early satiety) and/or loss of appetite, pelvic or abdominal pain or increased urinary urgency and/or frequency.

^†^Any one of pelvic abdominal pain or discomfort, loss of appetite, increase in abdominal size, able to feel a lump in the abdomen, or vaginal discharge.

Our estimates for the proportion of women aged 45–74 presenting over one year are shown in Table 3. According to the survival analysis 45.0% of women with a NICE guideline symptom in the reference week would not have had that same symptom within the previous 12 months (i.e. for 45.0% of those with a NICE symptom, at least one NICE symptom was new). Extrapolation yielded 11.9% (95% CI 5.0%, 18.3%) of all women aged 45–74 would present with a new NICE guideline symptom over one year. However, it should be noted that the estimates obtained from individual practices varied considerably.

**Table 3:** **Extrapolated estimates of the proportion of women aged 45–74 who would be offered symptoms-based ‘screening’ over 1 year according to different testing thresholds**

	**Percentage**	**95% CI**
Any ovarian cancer symptom	51.8%	44.0%-59.7%
Any NICE guideline symptom*	24.6%	17.4%-33.0%
Any new (in last year) NICE guideline symptom*^†^	11.9%	5.0%-18.3%
Any Index 2 symptom^‡^	20.3%	13.7%-28.5%
≥2 Index 2 symptoms^‡^	3.6%	1.0%-8.9%

NICE = National Institute for Health and Clinical Excellence, CI = confidence interval.

*Any one of persistent abdominal distension, feeling full (early satiety) and/or loss of appetite, pelvic or abdominal pain or increased urinary urgency and/or frequency.

^†^Estimated using Kaplan Meier.

^‡^Any one of pelvic abdominal pain or discomfort, loss of appetite, increase in abdominal size, able to feel a lump in the abdomen, or vaginal discharge.

Note: Extrapolations did not account for variation between practices in the proportion of women aged 45–74 with ≥1 ovarian cancer symptom. The estimated proportion of women presenting with an ovarian cancer symptom over one year varied from 32.2% (Woosehill Practice) to 69.1% (Chrisp Street Practice).

## Discussion

### Summary

According to our estimates, about a quarter of women aged 45–74 presenting to GP primary care would be offered symptoms-based screening for a NICE guideline symptom each year, however, this reduced to about a fifth when only ‘new’ symptoms were considered.

Despite having diverse locations, the four GP practices studied showed comparable proportions of women aged 45–74 who presented during the reference week. By contrast, the proportion of women in the target age range who presented with at least one ovarian cancer symptom relative to list size differed widely across surgeries.

### Strengths and limitations

A key strength of the study is that we collected data from a mixture of urban and non-urban practices to obtain a representative sample. In addition, having the last 10 consultations entries before the date of download provided information on symptom development. We used several testing scenarios to estimate the proportion of women who would be offered symptoms-based ovarian cancer screening, including an assessment of whether symptoms were new in the last year (testing is unlikely to be offered for longstanding symptoms). We used an age range that is most likely to be chosen for symptoms-based ovarian cancer screening; ovarian cancer incidence is much higher in postmenopausal women aged ≥45 and screening is not usually offered to those aged over 74. Finally, data were collected before initiatives to raise symptom awareness and encourage early presentation were commonplace (e.g. National Awareness and Early Diagnosis Initiative - NAEDI).

The main limitations are that the study was small and only records from women consulting during a single week were examined. This period is too short to take seasonal variations in consultations (e.g. flu season) into account. Additionally, we only studied four GP practices and these were seen to be heterogeneous. Another drawback is our inability to accurately assess other factors which would inform the decision to offer screening to women, such as symptom frequency, severity and persistence. In particular this could have led to overestimates of the proportion that would be offered symptoms-based screening.

### Comparison with existing literature

Estimates from a recent UK study [18] were smaller than ours. The authors used logistic regression to derive three different weighted ovarian cancer scores were derived using GP medical record data from 212 women with ovarian cancer and 1060 age-matched controls. Seven symptoms were included in each of the scores: bloating, urinary frequency, rectal bleeding, postmenopausal bleeding, loss of appetite, abdominal pain and abdominal distension. The main score had a specificity of 91.3% which is equivalent to 8.7% of women without ovarian cancer testing positive on the score over one year.

A prospective study randomised GP practices to being able to refer (or not refer) women aged ≥45 with any one of a list of 19 symptoms potentially related to ovarian cancer for immediate CA125 testing and transvaginal ultrasound [19]. Compliance was poor and only 39 out of 79 practices made referrals during the study period. Referral patterns were also extremely variable between GPs. Over the recruitment period that spanned almost 2½ years, only 317 women were referred.

### Implications for research/practice

Based on our findings, symptoms-based screening would result in roughly the same amount of screening as two yearly population screening for any ovarian cancer symptom, four yearly population screening for any NICE guideline symptom and eight yearly population screening for any ‘new’ NICE guideline symptom. One drawback of symptoms-based screening is that it is necessary to wait for symptoms to develop and for women to present. Lower socioeconomic status is associated with delayed presentation for some cancers [20], therefore, symptoms-based screening could lead to an increase in the social inequalities that already exist in ovarian cancer [21].

Nevertheless, current referral criteria for rapid access gynaecological-oncology clinics (relevant to ovarian cancer) only include two symptoms: suspicious pelvic mass and postmenopausal bleeding (if not on hormone replacement therapy). Symptoms-based screening could be a useful tool when other typical ovarian cancer symptoms are present but the index of suspicion is not sufficient to prompt referral or more expensive investigations. This could be particularly appealing given that the effectiveness of population-based ovarian cancer screening remains unclear. UKCTOCS (United Kingdom Collaborative Trial of Ovarian Cancer Screening) is the largest randomised trial of ovarian screening to date. Around 200,000 women from the general population were randomised to either screening with transvaginal ultrasound (TVS) or multimodal screening with CA125 and TVS or no screening. Data from the prevalent screening round found that sensitivity for primary invasive epithelial ovarian/tubal cancer was 89.5% and specificity was 99.8% in the multimodal arm [22]. Although these data are encouraging, the final picture will need to be balanced against unnecessary surgeries, complications and most importantly, needs to be able to demonstrate a mortality benefit. Mortality data are due in 2015 and are expected to provide major insights into ovarian screening.

Although our estimates for women who would require symptoms-based testing are relatively large, the extrapolations over one year are somewhat crude. Furthermore, any testing threshold for symptoms-based screening is likely to include a criterion for symptom persistence which was unaccounted for in this analysis. The NICE guidelines [14] and the symptom index produced by Goff *et al*. [23] stipulate that symptoms should occur more than 12 times per month Applying similar criteria should further reduce the number of women who require testing, as would the use of a score or similar. In this study, only 3.6% (95% CI 1.0%-8.9%) of women aged 45–74 would have had testing in one year if the requirement was for two or more Index 2 symptoms. Additionally, testing would not be offered to women who have already been recently tested (say the last 6–12 months) which would also reduce testing numbers.

## Conclusions

Over one year, between 11.9%-51.8% of women aged 45–74 who present to primary care would be offered symptoms-based ovarian screening depending on the guidelines adopted. Although there are other issues to consider, these data are encouraging since the numbers are considerably less than those required for annual (or even biennial) population screening and in reality, stricter criteria would be applied which would further reduce these figures.

## Abbreviations

EMIS: Egton Medical Information Systems
GP: General practitioner
NICE: National Institute for Health and Clinical Excellence
OC: Ovarian cancer
UK: United Kingdom
UKCTOCS: United Kingdom Collaborative Trial of Ovarian Cancer
